# Genome-wide analysis of the UGT gene family in *Rhododendron* and transcriptional correlation of *RiUGT43* with anthocyanin accumulation

**DOI:** 10.3389/fpls.2026.1957991

**Published:** 2026-09-23

**Authors:** Xingyun Zhong, Gaoyuan Hu, Yuhang Jiang, Haichao Hu, Xiaohong Xie, Yangshengkai Xu, Jiayi Wu, Jing Liu, Yueyan Wu, Pei Shi

**Affiliations:** College of Biology and Environment, Zhejiang Wanli University, Ningbo, China

**Keywords:** anthocyanin accumulation, petal coloration, *Rhododendron*, UDP-glycosyltransferase, UGT gene family

## Abstract

**Background:**

UDP-glycosyltransferases (UGTs) catalyze the glycosylation of anthocyanins and are key enzymes affecting anthocyanin stability and petal coloration. However, the evolutionary characteristics of the UGT family and its regulatory roles in flower color development remain poorly understood in woody ornamental plants.

**Methods:**

In this study, we identified 137 UGT genes in the *Rhododendron simsii* genome. Of these, 126 were unevenly distributed across the 13 chromosomes, whereas the remaining 11 were located on unanchored scaffolds. These genes were classified into 15 distinct subfamilies (designated A-O), among which subfamilies A and E contained the most members. Bioinformatic analyses of their physicochemical properties, conserved motifs, chromosomal locations and cis-acting regulatory elements revealed substantial structural and potential regulatory diversification within the *Rhododendron* UGT family. Integration of genome-family analysis with transcriptomic data from white, pink and red petals of *Rhododendron indicum* 'Huazhui' identified *RiUGT43* as a candidate associated with flower-color variation.

**Results:**

The *R. simsii* gene *RsUGT43* falls into group E based on the current phylogeny, while its homolog *RiUGT43* shows preferential expression in flowers. *RiUGT43*-GFP fluorescence and Western blot analysis in *Nicotiana benthamiana* leaves confirmed the fusion protein's cytoplasmic and nuclear localization and accumulation in this heterologous host. Changes in *RiUGT43* transcript abundance were accompanied by corresponding changes in anthocyanin content.

**Conclusion:**

In conclusion, our analysis of the UGT gene family in *Rhododendron* identified *RiUGT43* as a gene transcriptionally associated with anthocyanin accumulation, provided correlative evidence for flower color regulation in woody plants, and established a foundation for future functional studies of UGT-mediated anthocyanin regulation in woody plants.

## 1 Introduction

Flower color is one of the key traits determining the ornamental and economic value of plants and the development of novel flower colors remains a major objective and challenge in ornamental plant breeding (Gachon et al., 2005; Jones and Vogt, 2001; Tiwari et al., 2016).Flower pigmentation is a complex biological process determined by genetic, physiological and biochemical factors and is also influenced by environmental conditions, such as temperature and light. Anthocyanins are water-soluble flavonoid pigments whose composition, abundance and chemical modification contribute to red, purple and blue coloration in flowers, fruits and vegetative tissues (Passeri et al., 2016; Winkel-Shirley, 2001). Anthocyanins are synthesized through the phenylpropanoid and flavonoid biosynthetic pathways, which involve a series of structural enzymes, including chalcone synthase (*CHS*), chalcone isomerase (*CHI*), flavanone 3-hydroxylase (*F3H*), dihydroflavonol 4-reductase (*DFR*), anthocyanidin synthase (*ANS*) and UDP-dependent glycosyltransferases (*UGTs*) (Wang et al., 2025; Xu et al., 2015).

UGTs catalyze one of the final modification steps in anthocyanin biosynthesis by transferring sugar moieties from UDP-activated sugar donors to anthocyanidins, commonly at the 3-O or 5-O position (Gachon et al., 2005; Yamazaki et al., 1999). Glycosylation improves the water solubility and chemical stability of anthocyanins and facilitates their transport and accumulation in plant cells. Plant UGT proteins generally contain a highly conserved C-terminal plant secondary product glycosyltransferase box, known as the PSPG box. This domain comprises approximately 44 amino acids and is mainly involved in the recognition and binding of UDP-sugar donors. The plant UGT family was first systematically characterized in *Arabidopsis thaliana*, in which its members were initially classified into 14 subfamilies (Jackson et al., 2001; Li et al., 2001; Ma et al., 2025). Subsequent studies identified additional groups in other plant species, expanding the classification of plant UGTs to groups A-Q. Genome-wide analyses of the UGT family have since been conducted in numerous species, including maize (*Zea mays*) and tobacco (*Nicotiana tabacum*) (Li et al., 2001; Sun et al., 2026). However, the number and composition of UGT groups vary among plant species indicating lineage-specific expansion and functional diversification of this gene family.

Increasing evidence has demonstrated that UGT genes play important roles in anthocyanin modification, accumulation and plant coloration. Transcriptomic and metabolomic analyses have facilitated the identification of UGT genes associated with anthocyanin biosynthesis in various plant species, including purple tea (*Camellia sinensis*), ramie (*Boehmeria nivea*), and *Catalpa* (*Catalpa bungei*) (Chen et al., 2026; Feng et al., 2021; Maritim et al., 2021). Functional studies using virus-induced gene silencing (VIGS) or transient overexpression assays have further demonstrated the roles of several UGT genes in anthocyanin accumulation, such as *UGT73A93* in *Dianthus caryophyllus* L, *MwUGT2* in *Meconopsis*, *PhUGT78A22*, *PsUGT78A27*, and *PsUGT75L45* in *Paeonia suffruticosa*, *MtUGT84A1* in *Medicago truncatula*, *MrUGT78A26* and *MrUGT72B67* in *Morella rubra*, and *UGT71K3* in strawberry (*Fragaria* × *ananassa*) (Li et al., 2022; Lu et al., 2021; Ren et al., 2022; Song et al., 2016; Zhou et al., 2025, Zhou et al., 2026). Moreover, increasing evidence indicates that UGT genes are transcriptionally regulated by multiple transcription factors, including WRKY, bHLH, and MYB family members. For instance, pear UGTs are regulated by WRKY transcription factors, *IbUGT78D2* in purple sweet potato is controlled by *bHLH42*, and UGT expression in lilac is regulated by the SoMYB44 family (Cong et al., 2021; Hou et al., 2023; Wang et al., 2025). Nevertheless, the evolutionary characteristics and biological functions of UGT family members remain poorly understood in many plant species, particularly in woody ornamental plants.

*Rhododendrons* are species belonging to the genus *Rhododendron* in the family *Ericaceae*. Several anthocyanin-related glycosyltransferases have previously been characterized in *Rhododendron*. *Rd3GT1* and *Rd3GT6* from *R. delavayi* catalyze flavonoid 3-O-glycosylation and promote anthocyanin accumulation, whereas *Ra3GT2* from *R. agastum* glycosylates anthocyanidins and flavonols using UDP-glucose and UDP-galactose (Luo et al., 2025; Sun et al., 2022). These studies established the importance of individual 3-O-glycosyltransferases but did not provide a genome-wide framework for the diversity and evolution of the UGT family in *R. simsii*.

In this study, 137 *RsUGT* genes were identified from the genome of *R. simsii*. Bioinformatics analysis and spatiotemporal expression profiling revealed that *RiUGT43* was specifically highly expressed in flowers. Transient overexpression of *RiUGT43* in red flowers of *R. indicum* significantly increased total anthocyanin content, whereas VIGS-mediated silencing reduced anthocyanin accumulation. Subcellular localization showed that *RiUGT43* is localized to the cytoplasm and nucleus, suggesting a potential transcriptional association with flower color regulation. In summary, this study systematically analyzed the UGT gene family in *Rhododendron*, identified *RiUGT43* as a gene transcriptionally associated with anthocyanin accumulation, provided new insights into flower color regulation in woody plants, and established a foundation for elucidating the molecular mechanisms underlying flower color formation in *Rhododendron*.

## 2 Materials and methods

### 2.1 Plant materials

Perennial plants of *R. indicum* ‘Huazhui’ were maintained at the Wanjing *Rhododendron* Germplasm Nursery in Chaiqiao, Beilun District, Ningbo, China (121°27′40″–122°10′22″ E, 29°41′44″–29°58′48″ N). In spring 2025, petals from white, pink, and red flowers on the same plant were collected for transcriptome sequencing, with three biological replicates per color. In addition, roots, stems, leaves, and red petals at the closed-bud stage (S1), partially open stage (S2), full-bloom stage (S3), and early senescence stage (S4) were sampled from three to five uniformly growing plants. All samples were frozen in liquid nitrogen and stored at –80 °C until RNA extraction. *N. benthamiana* seedlings at 4–6 weeks of age were used for subcellular localization and Western blotting assays.

### 2.2 Identification and physicochemical characterization of the *RsUGT* gene family in *R. simsii*

To identify UGT gene family members in *R. simsii*, BLASTP searches were conducted using 112 previously reported *A. thaliana* UGT protein sequences as queries (Wang et al., 2025), combined with HMMER searches against the UGT conserved domain (PF00201) from Pfam, with an E-value threshold of ≤ 1 × 10⁻^5^. After removing redundant candidates, the integrity of the UGT domain was verified via InterPro and NCBI CDD, and genes lacking the complete domain were discarded. The remaining 137 genes were sequentially named *RsUGT1*–*RsUGT137* based on their chromosomal positions. Subsequently, physicochemical properties (amino acid length, molecular mass, theoretical pI, instability index, aliphatic index, and GRAVY) were calculated using ExPASy ProtParam, and subcellular localization was predicted by WoLF PSORT.

### 2.3 Retrieval of *RiUGT* homologues (*R. simsii* → *R. indicum*)

To obtain homologues of the 137 RsUGT proteins in *R. indicum* ‘Huazhui’, local BLASTP searches were performed against an *R. indicum* protein database using TBtools, with thresholds of ≥70% query coverage and ≥30% sequence identity. To validate the nomenclature, clarify the genomic correspondence of the target transcript, and examine its relationship with known flower color-related genes, multiple sequence alignment was performed using DNAMAN. Based on the alignment results, the transcript was designated according to its corresponding genomic homolog.

### 2.4 Phylogenetic analysis, conserved motif identification, and gene structure analysis

The full-length UGT protein sequences from *A. thaliana* and *R. simsii* were aligned using MEGA version 11.0. A phylogenetic tree was subsequently constructed using the neighbor-joining method with 1,000 bootstrap replicates to assess the reliability of the tree topology. The resulting phylogenetic tree was visualized and annotated using the Interactive Tree of Life (iTOL; https://itol.embl.de/).

Conserved motifs in the RsUGT protein sequences were identified using the MEME Suite (https://meme-suite.org/meme/tools/meme). The maximum number of motifs was set to 10, and the identified motifs were designated motif 1 to motif 10 according to the order generated by MEME. The genomic annotation file in GFF format was obtained from the *Rhododendron* Plant Genome Database. Information on the exon–intron organization and untranslated regions of the *RsUGT* genes was extracted from the annotation file. The gene structures, conserved motif distributions and phylogenetic relationships of the *RsUGT* family members were integrated and visualized using TBtools.

### 2.5 Chromosomal distribution and synteny analysis

The chromosomal distribution of the *RsUGT* genes was determined based on the reference genome and genome annotation files of *R. simsii*. Chromosome lengths and the physical coordinates of the *RsUGT* genes were extracted from the genome annotation file, and their chromosomal distribution was visualized using TBtools. Sequencing data from *R. indicum* were mapped to the *R. simsii* reference genome to establish the correspondence between *RiUGT* transcripts and their putative *RsUGT* homologues. Intraspecific collinearity and gene duplication analyses were performed to identify duplicated *RsUGT* gene pairs in the *R. simsii* genome. In addition, interspecific synteny analyses among *A. thaliana*, *R. simsii* and *Rhododendron ripense* were conducted using MCScanX. Syntenic blocks, homologous gene pairs and tandem duplication events were visualized using TBtools.

### 2.6 Analysis of *cis*-acting regulatory elements

Based on the *R. simsii* reference genome and its corresponding annotation file, the 2,000-bp genomic sequences upstream of the translation initiation codon (ATG) of each *RsUGT* gene were extracted using TBtools and regarded as putative promoter regions. The extracted sequences were submitted to the PlantCARE database (http://bioinformatics.psb.ugent.be/webtools/plantcare/html/) for the identification of putative *cis*-acting regulatory elements.

### 2.7 Transcriptome-based expression analysis of *RiUGT* genes

To examine the expression patterns of the *RiUGT* genes identified in Section 2.2, previously generated unpublished transcriptomic datasets from white, pink, and red flowers of *R. indicum* ‘Huazhui’ (three biological replicates per color group) were used. To enable cross-sample comparison of relative expression patterns while removing absolute abundance differences among genes, FPKM values were first extracted as normalized transcript abundance estimates, then transformed as log_2_ (FPKM), and finally standardized within each gene by row-wise z-score scaling across the nine samples. This scaled heatmap thus displays relative expression variation across flower colors for each gene, rather than absolute expression levels. Hierarchical clustering and heatmap visualization were performed using TBtools.

### 2.8 RNA extraction, cDNA synthesis, and qRT-PCR analysis

Total RNA was extracted from flowers of different colors (white, pink, and red) at the S3 stage (full-bloom stage), red flowers at the S1-S4 stages, as well as roots, stems, and leaves of *R. indicum* ‘Huazhui’ using a plant total RNA extraction kit (Vazyme, Nanjing, China). Approximately 0.1 g of each tissue sample was ground into powder, and RNA extraction was performed according to the manufacturer’s instructions. RNA concentration was measured as 100 ng·μL⁻¹. Total RNA (1 μg) was reverse transcribed in a 20-μL reaction using PrimeScript™ RT Master Mix (Takara, Tokyo, Japan). The resulting cDNA was used as a template for subsequent qRT-PCR analysis using NovoStart^®^ SYBR qRT-PCR SuperMix Plus (Novoprotein, Suzhou, China) on a Bio-Rad CFX96 real-time PCR system (Bio-Rad, Hercules, CA, USA). Each 10-μL reaction contained 5.0 μL of 2× NovoStart^®^ SYBR qRT-PCR SuperMix Plus, 0.5 μL of each forward and reverse primer (10 μmol L⁻¹), 1.0 μL of diluted cDNA template, and 3.0 μL of nuclease-free water. The amplification program consisted of an initial denaturation at 95 °C for 60 s, followed by 40 cycles of denaturation at 95 °C for 20 s and annealing at 60 °C for 60 s. *Actin* and *EF1α* served as reference genes, and the expression levels of the target genes were normalized against the geometric mean of the two reference genes. Relative transcript abundance was calculated using the 2^⁻ΔΔCt^ method and expressed as mean ± standard deviation (SD). Each sample was analyzed with three biological replicates and three technical replicates. The primers used in this study are listed in **Supplementary Table S2**.

### 2.9 Cloning and sequence characterization of *RiUGT43*

The physicochemical properties of *RiUGT43* were further analyzed using multiple bioinformatic tools. Using the transcriptome-derived *RiUGT43* sequence as a reference, gene-specific primers (listed in **Supplementary Table S2**) were designed to amplify the full-length coding sequence (CDS) of *RiUGT43* from first-strand cDNA (synthesized as described in Section 2.7). PCR was performed in a 50 μL reaction containing 2× Phanta Flash Master Mix (Dye Plus) and forward and reverse primers. The amplified product was purified and verified by Sanger sequencing.

### 2.10 Construction of recombinant vectors

For transient overexpression and subcellular localization analyses, the coding sequence of *RiUGT43* without the termination codon was amplified using gene-specific primers and inserted in-frame upstream of the enhanced green fluorescent protein (EGFP) gene in the pCAMBIA1300-35S-EGFP vector, generating the pCAMBIA1300-35S-*RiUGT43*-EGFP construct. For *virus*-induced gene silencing, a 300-bp gene-specific fragment of *RiUGT43* was amplified and inserted into the pTRV2 vector to generate the pTRV2-*RiUGT43* silencing construct. All recombinant plasmids were verified by Sanger sequencing, and all primers used for vector construction are listed in **Supplementary Table S2**.

### 2.11 Subcellular localization of *RiUGT43*

The sequence-verified recombinant plasmid pCAMBIA1300-35S-*RiUGT43*-EGFP and the corresponding empty vector were respectively transformed into *Agrobacterium tumefaciens* strain GV3101 competent cells. Positive single colonies were selected and cultured in LB liquid medium containing KR antibiotics (kanamycin and rifampicin) at 28 °C. The bacterial cells were harvested by centrifugation at 8000 r/min for 60 s, resuspended in infiltration buffer (10 mM MES, pH 5.6, 10 mM MgCl_2_, 200 μM acetosyringone), and adjusted to an OD_600_ of 0.8. The *Agrobacterium* suspensions carrying the recombinant plasmid, or the empty vector were separately infiltrated into the leaves of *N. benthamiana*. The infiltrated plants were incubated at 25 °C for 72 h. EGFP fluorescence was observed at 48 h post-infiltration using a confocal laser scanning microscope (Leica Microsystems, Germany).

### 2.12 Western blot analysis

Total proteins were extracted from *N. benthamiana* leaves infiltrated with *A. tumefaciens* carrying pCAMBIA1300-35S-*RiUGT43*-EGFP or the empty vector. Protein samples (20 μg per lane) were denatured, separated by 10% SDS-PAGE, and wet-transferred onto a nitrocellulose membrane. After blocking with 5% non-fat milk in PBST, the membrane was incubated with mouse anti-GFP monoclonal antibody (diluted 1:5000) at 4 °C overnight, followed by HRP-conjugated goat anti-mouse secondary antibody (diluted 1:10000). Protein signals were detected using an enhanced chemiluminescence (ECL) substrate and captured with a VersaDoc MP 4000 imaging system.

### 2.13 Transient expression, VIGS, and anthocyanin determination

For transient overexpression, fully opened red petals of *R. indicum* ‘Huazhui’ at the full-bloom stage were infiltrated with Agrobacterium suspension carrying pCAMBIA1300-35S-*RiUGT43*-EGFP, with the empty vector serving as the control. For *virus*-induced gene silencing (VIGS), equal volumes of *Agrobacterium* suspensions carrying pTRV1 and pTRV2-*RiUGT43* were mixed at a 1:1 ratio and infiltrated into the petals, while a mixture of pTRV1 and empty pTRV2 was used as the negative control. After infiltration, the flowers were kept in darkness for 3 d, after which the petals were collected. A portion of the petals was photographed to document changes in coloration, and the remaining petals were immediately frozen in liquid nitrogen and stored at −80 °C.

Total anthocyanins were extracted using acidified ethanol (Yang et al., 2026). Frozen petal powder (0.1 g) was extracted with 2 mL of 70% ethanol containing 0.1% HCl, vortexed, and sonicated at 40 °C in darkness for 1 h. After centrifugation at 8000 r/min for 10 min at 4 °C, the supernatant was collected. The extract was diluted with KCl buffer (pH 1.0) and sodium acetate buffer (pH 4.5), equilibrated in darkness for 20 min, and the absorbance was measured at 530 nm and 700 nm using a SpectraMax 190 microplate reader (Molecular Devices, Sunnyvale, CA, USA). Corrected absorbance was calculated as A = (A_530_ − A_700_)pH_1.0_ − (A_530_ − A_700_) pH_4.5_ (Yang et al., 2026). Total anthocyanin content was expressed as mg cyanidin-3-O-glucoside equivalents per 100 g fresh weight, using a molecular weight of 449.2 g·mol⁻¹ and a molar extinction coefficient of 26,900 L·mol⁻¹·cm⁻¹ (Yang et al., 2026). The transcript levels of *RiUGT43* were determined by qRT-PCR. Three independent biological replicates were performed for each treatment, and the primers used are listed in **Supplementary Table S2**.

### 2.14 Statistical analysis

All statistical analyses were performed using SPSS (IBM, Chicago, USA, v27.0.1). For multi-group comparisons, one-way ANOVA was used, and when significant, means were separated by Duncan’s multiple range test at *P* < 0.05 (two-tailed). For two-group comparisons, significance was determined by two-tailed Student’s *t*-test, with significance levels indicated as ***P < 0.001 and ****P < 0.0001. For qRT-PCR datasets, statistical tests were performed on ΔCt values (technical triplicates averaged; *n* = 3 biological replicates) prior to fold-change transformation. All data are reported as mean ± SD of three independent biological replicates (technical triplicates averaged prior to analysis). Graphs were produced using GraphPad Prism (v9.5.0, GraphPad Software, San Diego, CA, USA), and figure panels were assembled in Adobe Illustrator (v28.6, Adobe Inc., San Jose, CA, USA).

## 3 Results

### 3.1 Physicochemical characterization of the *RsUGT* proteins

The UGT family in *R. simsii* comprised 137 members, which were sequentially designated as *RsUGT1* to *RsUGT137* according to their chromosomal locations. The physicochemical properties of these RsUGT proteins were as follows: predicted protein lengths ranged from 129 to 1,260 amino acids (mean: 490.42 aa), molecular weights from 13.90 to 140.85 kDa, and theoretical pI values from 4.87 to 9.49. All RsUGT proteins exhibited instability indices above 40 and negative GRAVY values, indicating that they are unstable and hydrophilic proteins. Subcellular localization predictions suggested that all RsUGT proteins are localized in the cytoplasm. Detailed physicochemical properties are provided in **Supplementary Table S1**.

### 3.2 Phylogenetic analysis of the *RsUGT* gene family in *R. simsii*

To further investigate the evolutionary relationships of the *RsUGT* gene family, a neighbor-joining (NJ) phylogenetic tree was constructed using 137 identified RsUGT protein sequences and 112 previously reported UGT protein sequences from *A. thaliana* (**Figure 1**). Based on the branching pattern of the phylogenetic tree, all analyzed UGT proteins were classified into 15 subfamilies (designated A-O), among which subfamily E contained the largest number of members, followed by subfamily A. Groups P and Q were not recovered in the present analysis because no corresponding *A. thaliana* reference sequences were available for these two groups (**Figure 1**).

**Figure 1:**
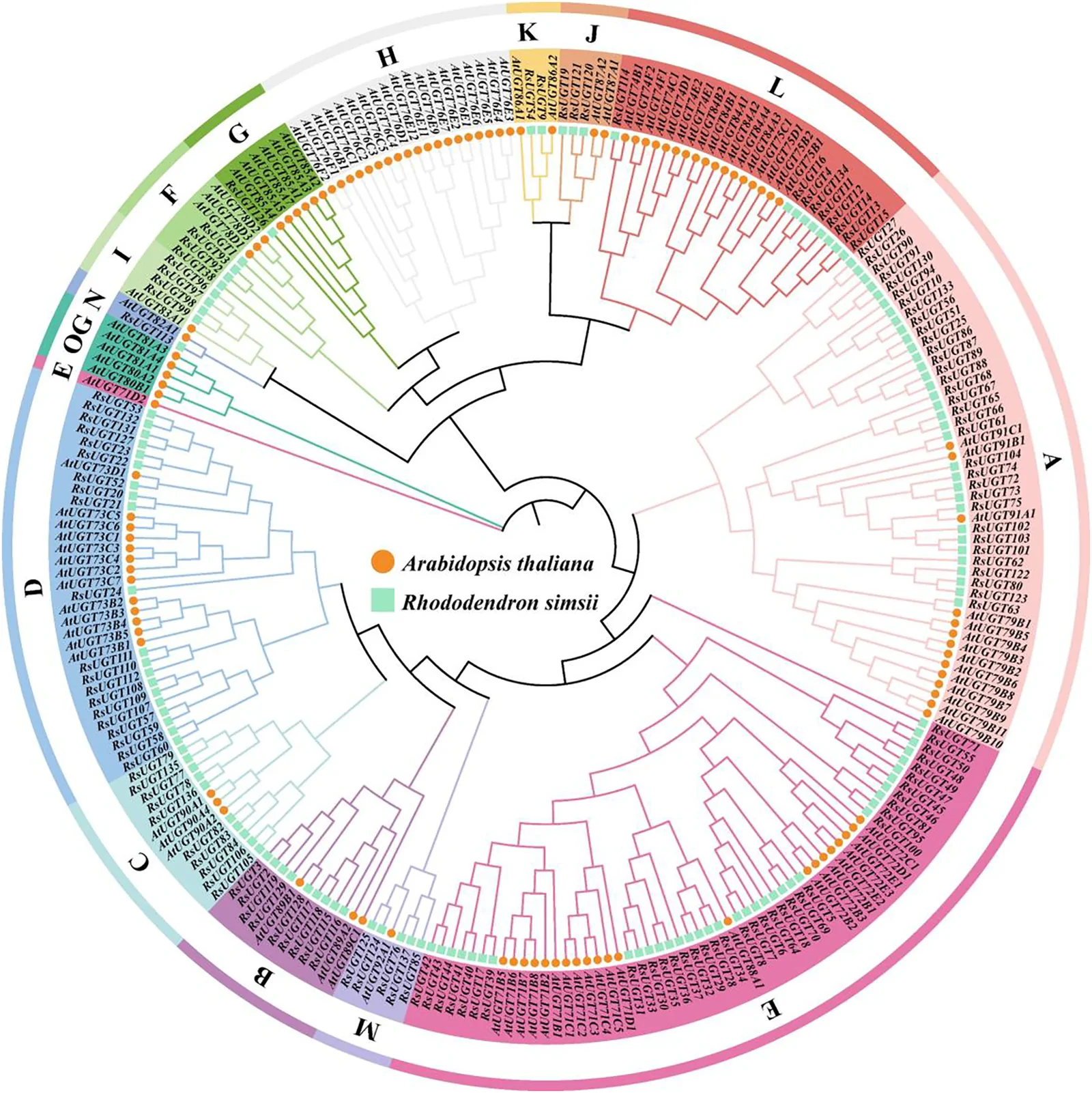
Phylogenetic relationships and subfamily classification of UGT proteins from *R. simsii* and *A. thaliana*. The phylogenetic tree was constructed using the full-length amino acid sequences of 137 UGT proteins from *R. simsii* (*Rs*, *n* = 137) and 112 UGT proteins from *A. thaliana* (*At*, *n* = 112) using the neighbor-joining method implemented in MEGA. Branch support was assessed using 1,000 bootstrap replicates. The colored sectors represent 15 UGT subfamilies, designated groups A–O. Orange circles and green squares in the inner ring indicate UGT proteins from *A. thaliana* and *R. simsii*, respectively.

Among the 15 subfamilies, *RsUGT43* was identified as a member of subfamily E. Multiple sequence alignment further revealed that *RsUGT43* possesses the same conserved UGT domains as the functionally characterized *A*t*UGT79B2* and *AtUGT79B3*, both of which have been verified to participate in anthocyanin accumulation and flower coloration (**Supplementary Figure S5**).

### 3.3 Gene structure and conserved motif analyses of the *RsUGT* gene family

Structural analysis revealed that *RsUGT* genes exhibit structural diversity: most members contain two or more exons (i.e., containing introns), while a few are single-exon genes (lacking introns). Within the same clade, most members exhibited similar lengths and identical exon–intron distribution patterns. In subfamily E, most members contained 1–2 exons, with only a few possessing multiple exons; the vast majority showed no UTR regions in the predicted gene models, whereas only a small number contained them. Furthermore, all RsUGT proteins contained Motif 1 and Motif 2, and within the same subfamily, the types, numbers, and arrangement patterns of conserved motifs were largely consistent (**Figure 2**). These structural features indicate that the *RsUGT* gene family is highly conserved in its structural architecture.

**Figure 2:**
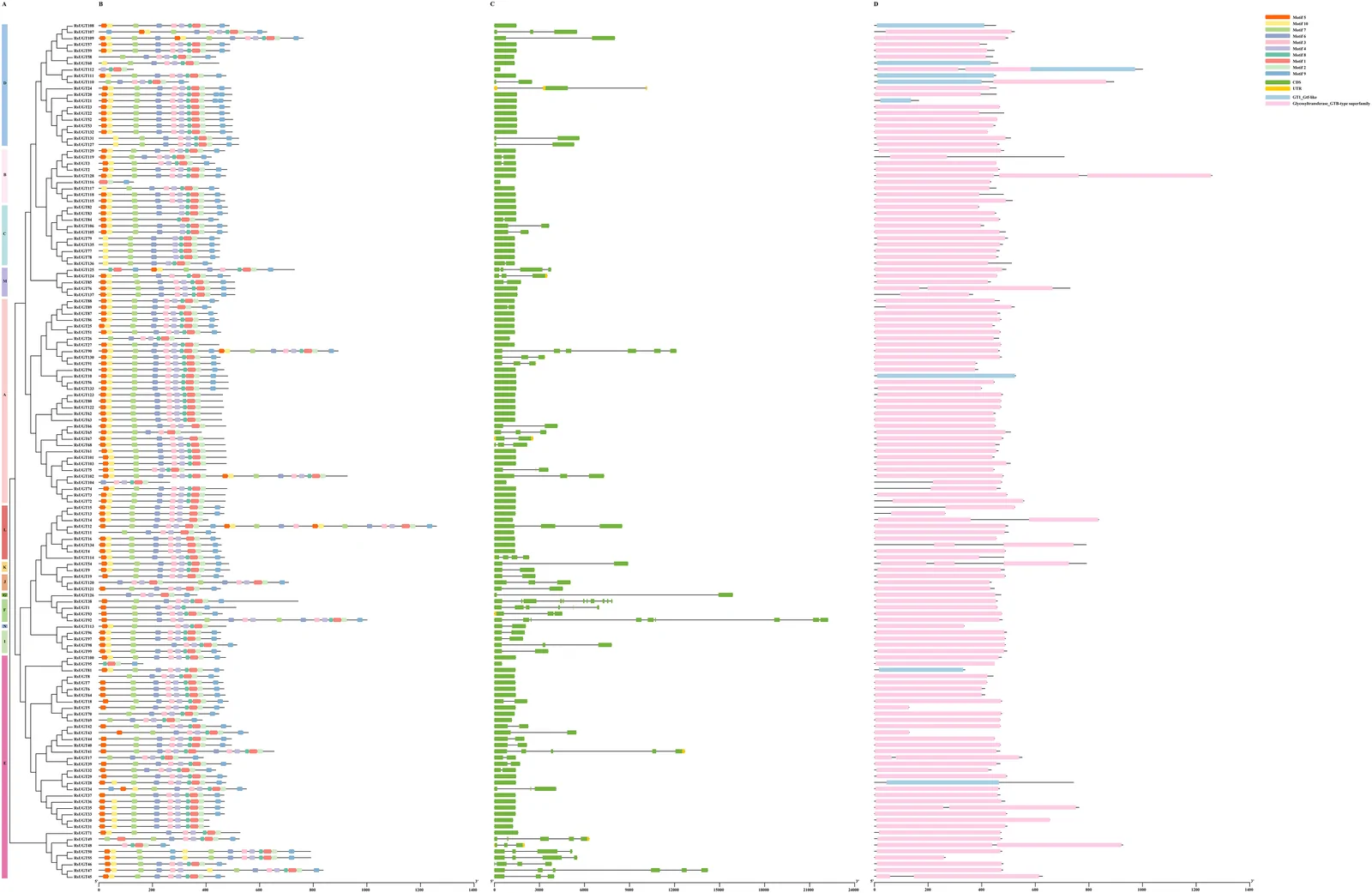
Phylogenetic relationships, conserved motifs, conserved domains, and gene structures of the *RsUGT* family. **(A)** Neighbour-joining phylogenetic tree constructed using the full-length amino acid sequences of RsUGT proteins. The tree is colored according to subfamily classification, with different colors representing distinct evolutionary groups. **(B)** Distribution of conserved motifs predicted using MEME. **(C)** Exon–intron structures inferred from alignments of coding sequences against the corresponding genomic sequences. Boxes and lines represent exons, introns, and untranslated regions (UTRs), as indicated in the legend. **(D)** Conserved domain composition identified using the NCBI Conserved Domain Database.

### 3.4 Chromosomal distribution and synteny analysis of the *RsUGT* genes

To investigate the genomic organization of the UGT gene family in *R. simsii*, the 137 identified *RsUGT* genes were mapped to the *R. simsii* genome (**Figure 3**). Among them, 126 *RsUGT* genes were successfully anchored to the 13 chromosomes, whereas the remaining 11 genes were located on unanchored genomic sequences and could not be assigned to any chromosome. The *RsUGT* genes were unevenly distributed across the 13 chromosomes. Chromosome 3 (Chr3) contained the largest number of *RsUGT* genes, with 21 members accounting for 15.3% of the entire family, followed by chromosome 4 (Chr4), which contained 18 genes and accounted for 13.1% of the family. By contrast, chromosome 5 (Chr5) contained only one *RsUGT* gene, representing the chromosome with the fewest *RsUGT* members.

**Figure 3:**
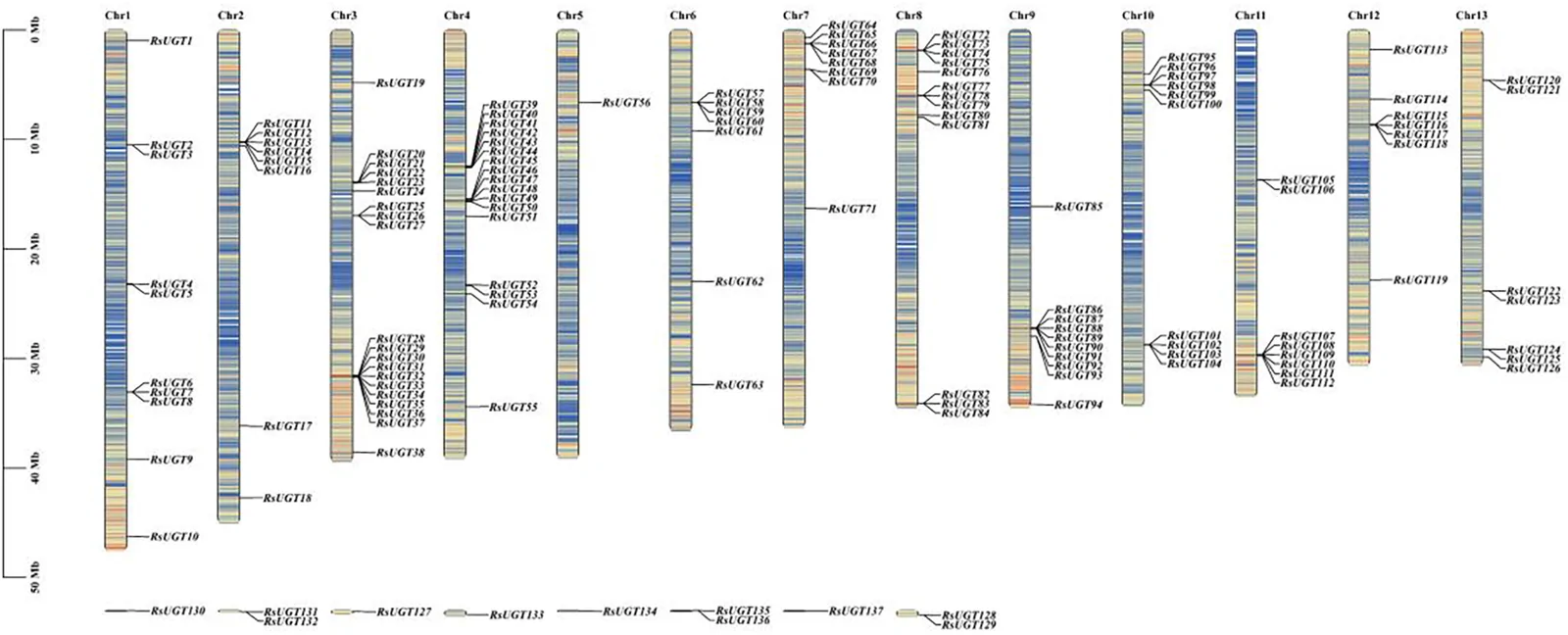
Chromosomal distribution of the *RsUGT* genes in *R. simsii*. The physical positions of the *RsUGT* genes were obtained from the genome annotation file and visualized using TBtools. The vertical bars represent the 13 chromosomes of *R. simsii*, with chromosome numbers (Chr) indicated at the top. The scale on the left indicates physical distance in megabases (Mb). Genes that could not be anchored to specific chromosomes are displayed at the bottom. In total, 126 *RsUGT* genes were mapped to the 13 chromosomes, whereas the remaining 11 genes were located on unanchored scaffolds.

Gene duplication is a major driving force underlying the evolution and expansion of gene families and generally occurs through tandem duplication or segmental duplication. To investigate the duplication patterns of the *RsUGT* family, intraspecific collinearity among the *RsUGT* genes was analysed. A total of nine collinear gene pairs were identified: *RsUGT1/RsUGT38*, *RsUGT5/RsUGT69*, *RsUGT6/RsUGT64*, *RsUGT22/RsUGT52*, *RsUGT61/RsUGT72*, *RsUGT61/RsUGT101*, *RsUGT72/RsUGT101*, *RsUGT81/RsUGT95*, and *RsUGT81/RsUGT100* (**Figure 4A**).

**Figure 4:**
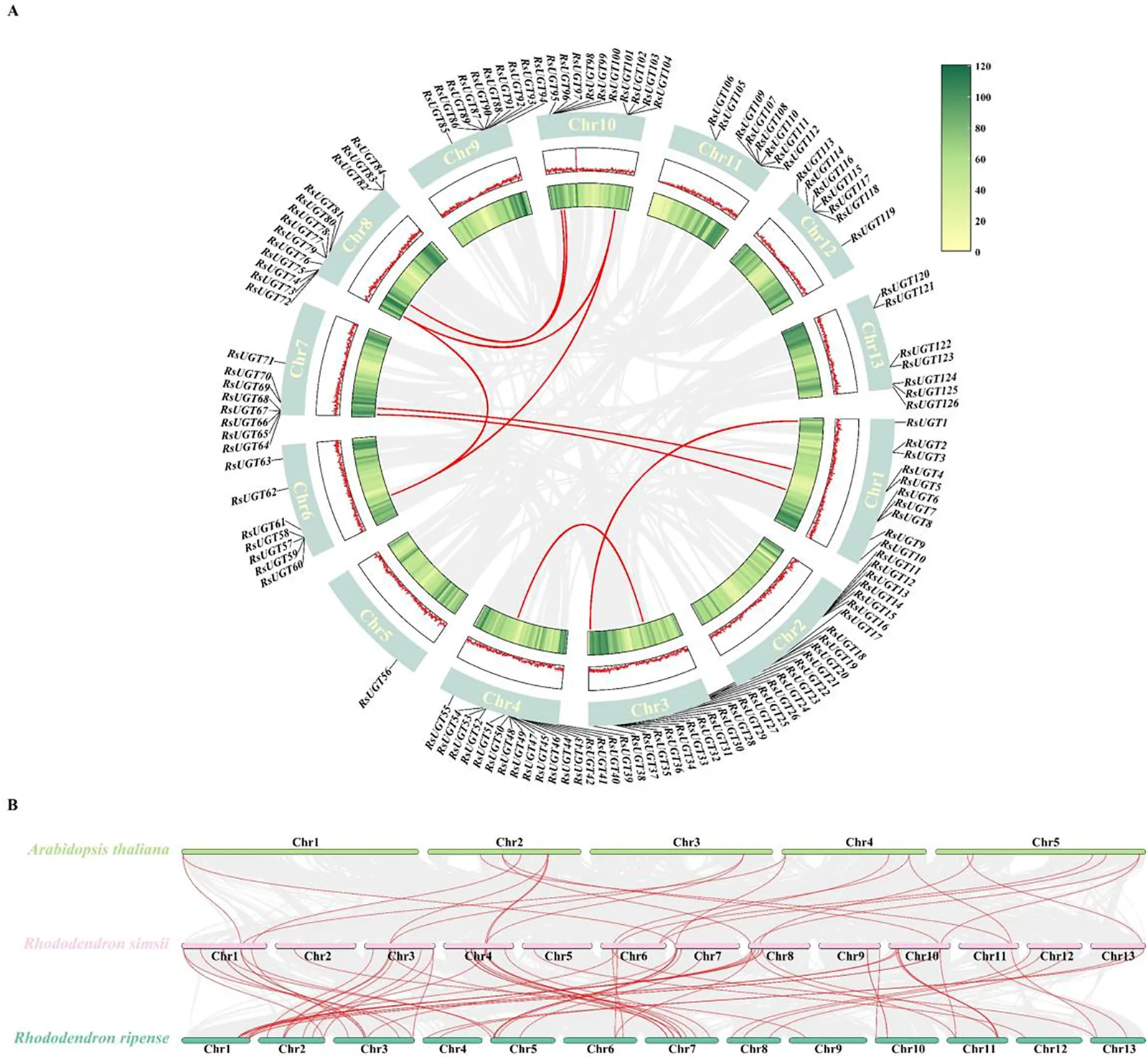
Chromosomal distribution and syntenic relationships of the *RsUGT* genes. **(A)** Intraspecific synteny analysis of the *RsUGT* gene family in *R. simsii*. **(B)** Interspecific synteny analysis of UGT genes among *A. thaliana*, *R. simsii*, and *R. ripense*.

To further explore the evolutionary history of the *RsUGT* family, interspecific synteny maps were constructed among *A. thaliana*, *R. simsii*, and *R. ripense*. A total of 22 syntenic UGT gene pairs were identified between *R. simsii* and *A. thaliana*, whereas 67 syntenic gene pairs were detected between *R. simsii* and *R. ripense*. Notably, 12 *RsUGT* genes showed syntenic relationships with homologous genes in both *A. thaliana* and *R. ripense*, suggesting that these genes may have been relatively conserved during the evolution of the UGT family (**Figure 4B**).

### 3.5 *Cis*-acting regulatory elements in the promoters of *RsUGT* genes

To investigate the transcriptional regulation of the *RsUGT* family, *cis*-acting regulatory elements in the 2,000-bp upstream promoter regions of 137 genes were analyzed. These elements were grouped into light-responsive, hormone-responsive, growth- and development-related, and biotic- and abiotic-stress-responsive elements (**Figure 5**). Hormone-responsive motifs were involved in methyl jasmonate, abscisic acid, auxin, gibberellin, and salicylic acid signaling. Light-responsive elements were present in all promoters, with the G-box and Box 4 being the most prevalent (94.16% and 81.02%, respectively), suggesting extensive regulation by light signals. Several promoters also contained development-related elements (e.g., meristem and seed-specific). Overall, the *RsUGT* promoters were dominated by light- and hormone-responsive elements, along with stress-related motifs, providing insights into their regulatory roles in anthocyanin metabolism and environmental adaptation.

**Figure 5:**
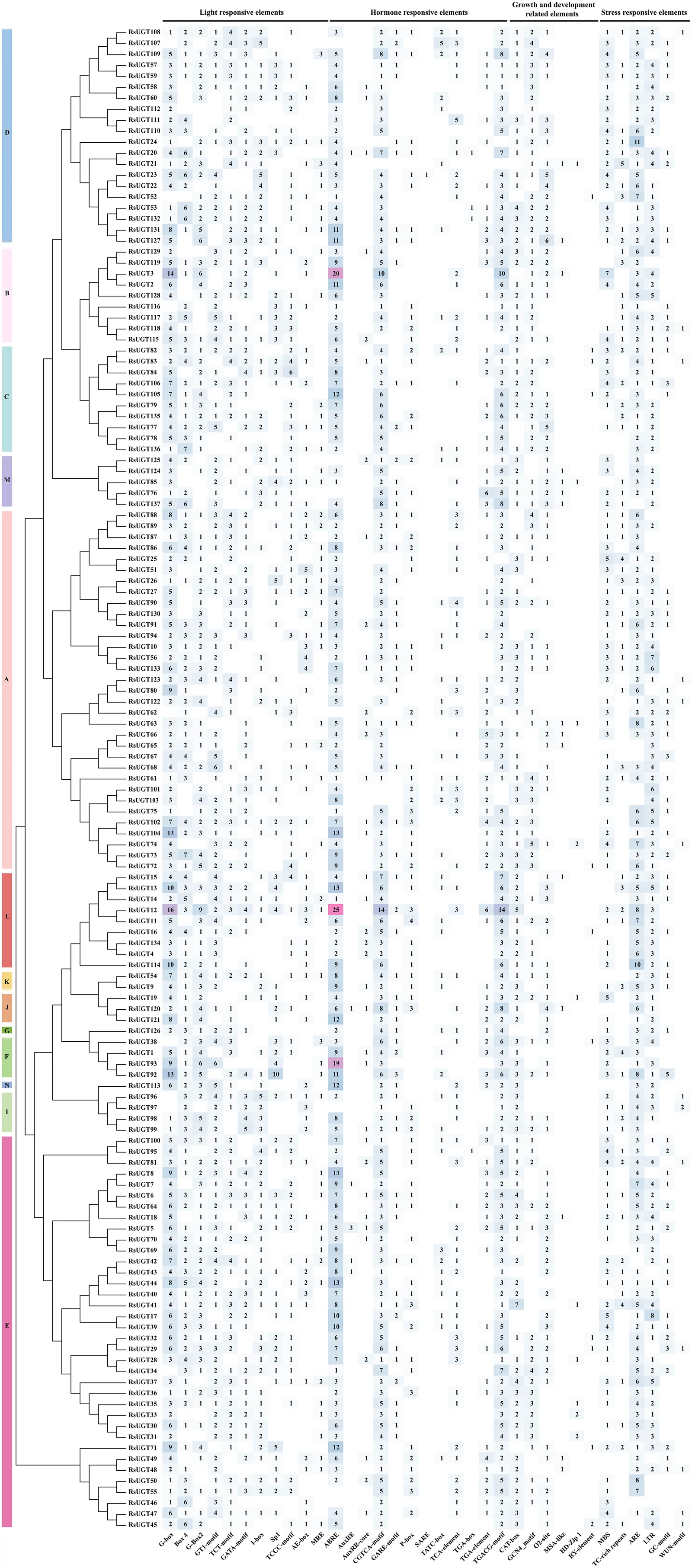
Distribution of *cis*-regulatory elements in the 2-kb promoter regions of 137 *RsUGT* genes from *R.simsii*. The first column on the left represents 15 phylogenetic subfamilies, and the bottom row indicates four functional categories of *cis*-elements: hormone-responsive, stress-responsive, light-responsive, and development-related elements.

### 3.6 Sequence and structural features of *RiUGT43*

To determine the genomic counterpart of *RiUGT43*, the deduced amino acid sequence of *RiUGT43* was aligned with those of *RsUGT42*, *RsUGT43*, and *RsUGT44*.*RiUGT43* shared the highest sequence identity with *RsUGT43* (95.7%), indicating that they are orthologous counterparts between the two *Rhododendron* species (**Supplementary Figure S1**).

Transmembrane domain prediction revealed no obvious transmembrane helices, consistent with its hydrophilic nature (**Supplementary Figure S2**). Secondary structure prediction indicated that *RiUGT43* is predominantly composed of alpha helices, random coils, and extended strands, with random coils being the most abundant and no beta sheets detected (**Supplementary Figure S3**). Additionally, *RiUGT43* contains a complete plant UGT signature PSPG box spanning residues 417-460, with the highly conserved HCGWNS motif located at residues 435-440 (**Supplementary Figure S4**).

### 3.7 Expression analysis of *RiUGT* genes across flower colors

A total of 48 UGT genes were identified in *R. indicum* ‘Huazhui’, from which 12 representative genes were selected to determine their expression levels in white, pink, and red flowers. The results showed that most genes were highly expressed in red flowers, while only a few were highly expressed in pink flowers (**Figures 6B–M**). Notably, *RiUGT43* was significantly enriched in red flowers, with an expression level approximately 20-fold higher than in white flowers, and its expression increased progressively with the deepening of flower color across white, pink, and red petals (**Figure 6E**). This pattern was highly consistent with its transcriptome FPKM data and hierarchical clustering analysis (**Figure 6A**). This suggests that *RiUGT43* transcript abundance was associated with red pigmentation, warranting further study.

**Figure 6:**
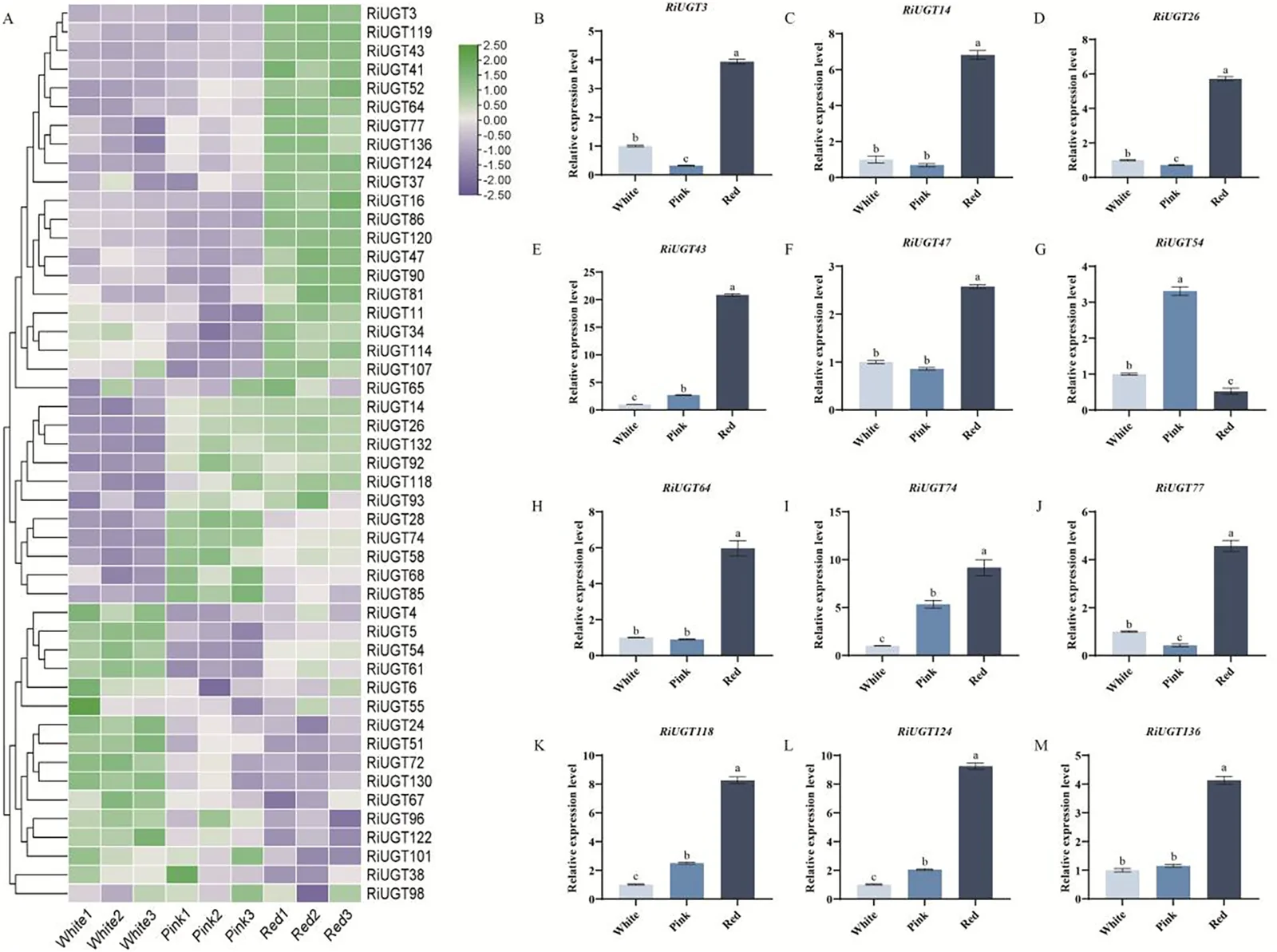
Expression profiles of *RiUGT* genes in white, pink, and red flowers of *R. indicum* ‘Huazhui’. **(A)** Heatmap of log_2_(FPKM) values for 48 *RiUGT* genes across three biological replicates of each flower-color group. A common color scale was applied across all genes and samples. **(B–M)** qRT-PCR validation of 12 candidate *RiUGT* genes in white, pink, and red flowers: **(B)** *RiUGT3*; **(C)** *RiUGT14*; **(D)** *RiUGT26*; **(E)** *RiUGT43*; **(F)** *RiUGT47*; **(G)** *RiUGT54*; **(H)** *RiUGT64*; **(I)** *RiUGT74*; **(J)** *RiUGT77*; **(K)** *RiUGT118*; **(L)** *RiUGT124*; and **(M)** *RiUGT136*. For each gene, expression levels were calculated relative to those in white flowers (calibrator, set as 1.0). Data are presented as mean ± SD of three biological replicates (n = 3). Different lowercase letters indicate significant differences among flower-color groups (one-way ANOVA with Duncan’s multiple range test, *P* < 0.05).

### 3.8 Spatiotemporal expression patterns of *RiUGT43*

To investigate the potential role of *RiUGT43* in flower color development, its expression patterns across different tissues and floral developmental stages were examined in the red-flowered *R. indicum* ‘Huazhui’ using qRT–PCR. During flower opening, *R. indicum* ‘Huazhui’ exhibited distinct developmental changes in floral morphology (**Figure 7A**). The transcript abundance of *RiUGT43* varied markedly across the four floral developmental stages (**Figure 7C**). Expression increased during flower development and reached its highest level at the full-bloom stage (S3), at which it was approximately 4.8-fold higher than that at the closed-bud stage (S1). Tissue-specific expression analysis further revealed that *RiUGT43* was expressed at very low levels in the vegetative organs, including roots, stems and leaves, whereas substantially higher transcript abundance was detected in flowers (**Figures 7B, D**). The preferential expression of *RiUGT43* in floral tissues, together with its developmental-stage-dependent expression pattern, suggests that this gene may be transcriptionally associated with flower pigmentation in *R. indicum* ‘Huazhui’.

**Figure 7:**
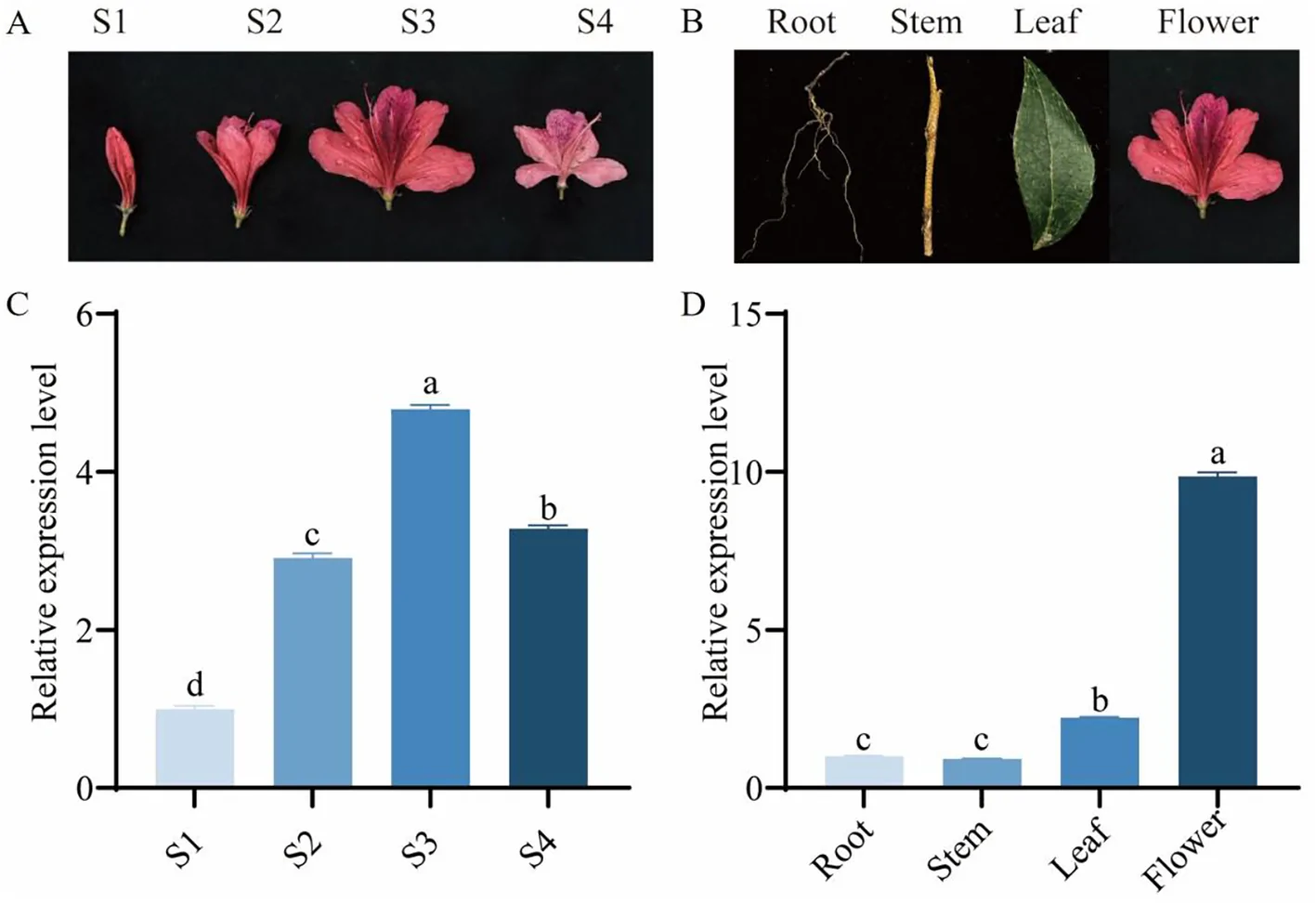
Spatiotemporal expression patterns of *RiUGT43* in *R. indicum* ‘Huazhui’. **(A)** Representative phenotypes of red flowers at four developmental stages: S1, closed-bud stage; S2, half-open stage; S3, full-bloom stage; and S4, early-senescence stage. **(B)** Representative phenotypes of roots, stems, leaves, and flowers. **(C)** Relative expression levels of *RiUGT43* at four floral developmental stages determined by qRT-PCR. **(D)** Relative expression levels of *RiUGT43* in roots, stems, leaves, and flowers determined by qRT-PCR. Data are presented as mean ± SD of three biological replicates (n = 3). Different lowercase letters indicate significant differences among groups (one-way ANOVA with Duncan’s multiple range test, *P* < 0.05).

### 3.9 Subcellular localization and expression analysis of *RiUGT43*

A schematic representation of the constructed pCAMBIA1300-*RiUGT43*-EGFP fusion vector is shown in **Figure 8A**. Heterologous expression of this construct in epidermal cells of *N. benthamiana* revealed that the *RiUGT43*-EGFP fusion protein was localized to both the cytoplasm and the nucleus (**Figure 8B**). Western blotting with an anti-GFP antibody further confirmed the accumulation of the fusion protein in infiltrated tobacco leaves (**Figure 8C**).

**Figure 8:**
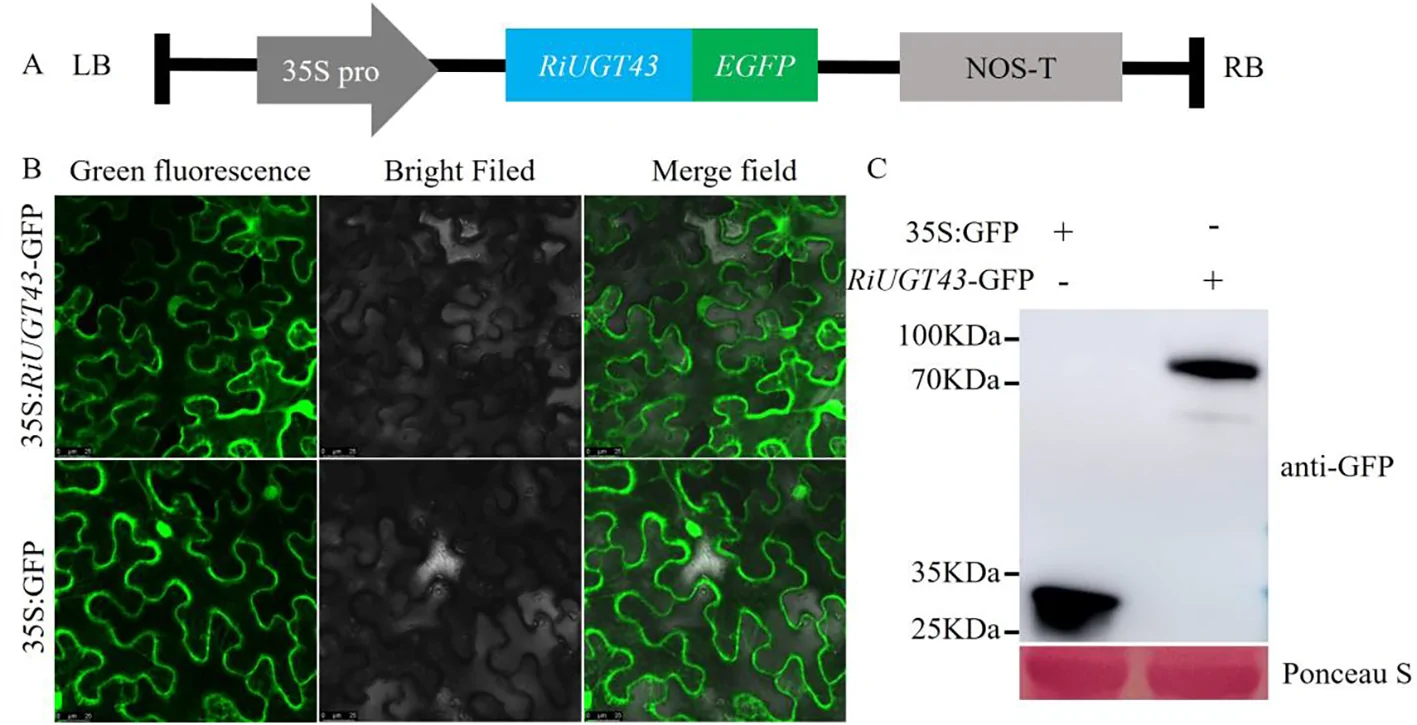
Subcellular localization and expression verification of *RiUGT43*. **(A)** Schematic diagram of the pCAMBIA1300-*RiUGT43*-EGFP construct, containing the 35S promoter, *RiUGT43*, EGFP, and NOS terminator, flanked by LB and RB. **(B)** The pCAMBIA1300-*RiUGT43*-EGFP construct was transiently expressed in *N. benthamiana* epidermal cells. Fluorescence microscopy showed that the fusion protein was present in both the cytoplasm and the nucleus. **(C)** Western blotting of total protein extracts from *N. benthamiana* leaves using an anti-GFP antibody confirmed the expression of the *RiUGT43*-GFP fusion protein, with a band detected at approximately 70 kDa. Scale bar, 25 μm.

### 3.10 Transcriptional correlation of *RiUGT43* with anthocyanin accumulation by transient overexpression and virus-induced gene silencing

Transient overexpression and gene silencing assays were performed in the red flowers of *R. indicum* ‘Huazhui’. qRT-PCR analysis revealed that the transcript abundance of *RiUGT43* in overexpression petals increased by 20.5-fold compared with the empty vector control (****P < 0.0001; **Figure 9C**). Correspondingly, overexpression of *RiUGT43* increased the anthocyanin content to 1.48-fold of the control, corresponding to a 48% increase (****P < 0.0001; **Figure 9E**). In the silencing assay, the transcript abundance of *RiUGT43* in silenced petals decreased to 0.41-fold of the empty vector control (****P < 0.0001; **Figure 9D**), and suppression of *RiUGT43* decreased the anthocyanin content to 0.88-fold of the control, corresponding to a 12% decrease (***P < 0.001; **Figure 9F**).

**Figure 9:**
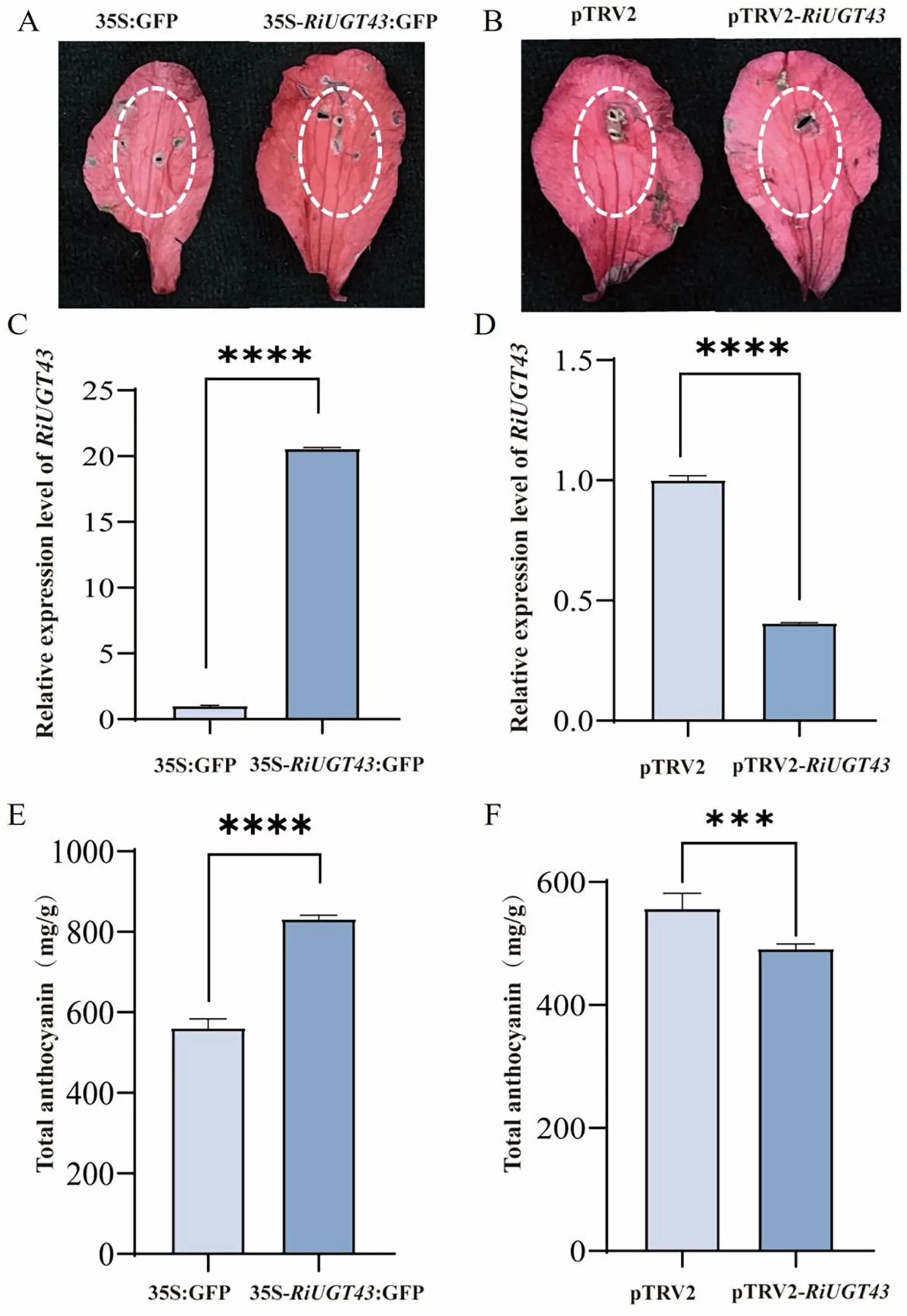
Association between *RiUGT43* transcript abundance and anthocyanin accumulation in *R. indicum* ‘Huazhui’. **(A, B)** Representative phenotypes of infiltrated petals. **(C)** qRT-PCR analysis of *RiUGT43* transcript levels in overexpression petals. **(D)** qRT-PCR analysis of *RiUGT43* transcript levels in silencing petals. **(E)** Total anthocyanin content in overexpression petals. **(F)** Total anthocyanin content in silencing petals. Data are presented as means ± SD from three independent biological replicates. Significant differences were determined by two-tailed Student’s *t*-test: ***p < 0.001, ****p < 0.0001.

In summary, anthocyanin accumulation was positively correlated with the expression level of *RiUGT43*; higher *RiUGT43* expression was associated with greater anthocyanin accumulation.

## 4 Discussion

In this study, 137 UGT genes were identified from *R. simsii*, revealing both evolutionary conservation and diversification of the plant UGT family. Expansion events were mainly concentrated in Groups A and E, which contained multiple duplicated gene pairs, suggesting that these groups may have undergone functional diversification during evolution. All *RsUGT* members exhibited similar exon–intron structures and conserved motif compositions, indicating their shared evolutionary origin.

Conserved motif analysis showed that all 137 RsUGT proteins contained the characteristic UGT conserved domain composed of Motif 1 and Motif 4, which has also been reported in strawberry UGTs. Gene expression regulation is often mediated through interactions between *cis*-acting elements and transcription factors. In the present study, multiple *cis*-acting regulatory elements were identified in the promoter regions of *RsUGT* genes, including light-responsive, hormone-responsive, and stress-responsive elements, among which light- and hormone-responsive elements were the most abundant. Previous studies have demonstrated that anthocyanin biosynthetic genes in tea plant (*Camellia sinensis* (L.) O. Ktze.) are induced by high light intensity, whereas weak light conditions suppress their expression. In peach, the flower-expressed genes *Prupe.1G091100* and *Prupe.1G091000* may participate in anthocyanin glycosylation, and *Prupe.1G091100* is also significantly induced by UV-B irradiation (Wu et al., 2017). In purple-leaf tea, *CsUGT72AM1* and *CsMYB6A* are highly co-expressed in purple leaves, and *CsUGT72AM1* catalyzes multiple flavonol substrates, indicating that UGT genes may be jointly regulated by light signaling and transcription factors and contribute to flavonoid accumulation. Similarly, UGT genes in apple (*Malus domestica* Borkh.) can also be regulated by MYB transcription factors (Guo et al., 2026; He et al., 2018). These findings suggest that the regulation of *Rhododendron* UGT genes, including *RiUGT43*, may involve complex regulatory networks integrating light signaling and transcription factors.

Phylogenetic analysis assigned *RiUGT43* (corresponding to *RsUGT43*) to Group E of the UGT family. Although this gene did not directly cluster with the functionally characterized anthocyanin-related *AtUGT79B2* and *AtUGT79B3* from *A. thaliana*, multiple sequence alignment revealed that they still retain the typical conserved motifs of the plant UDP-glycosyltransferase family. Previous studies have shown that *AtUGT79B2* and *AtUGT79B3* positively regulate anthocyanin accumulation to enhance tolerance to abiotic stresses in *A. thaliana*, indicating that both genes possess anthocyanin-related glycosyltransferase activities (Li et al., 2017). Notably, functional convergence within the UGT family has been documented, in which members from different phylogenetic branches participate in the same metabolic pathways. For example, although *AtUGT79B2* and *AtUGT79B3* (Group A) and *PpUGT78A2* (Group F) belong to different phylogenetic groups, both have been confirmed to be involved in anthocyanin glycosylation and accumulation, suggesting that the phylogenetic grouping of UGT genes does not strictly correspond to their biochemical functions (Cheng et al., 2014; Li et al., 2017). Given that *RiUGT43* shares the same UDP-glycosyltransferase domains with *AtUGT79B2* and *AtUGT79B3*, we propose that it may be transcriptionally associated with anthocyanin accumulation in *Rhododendron* petals, although this inference remains at the transcriptional level and does not confirm its enzymatic function. Direct protein-level evidence will be required in future studies to determine whether it has catalytic activity, and such investigations will be pursued with optimized experimental approaches.

The functional roles of UGT genes in anthocyanin biosynthesis have been validated in multiple plant species. In crabapple (*Malus spectabilis* (Ait.) Borkh.) and apple, UGT genes mainly catalyze the formation of cyanidin-3-*O*-galactoside, contributing to red coloration of fruit skin (Ma et al., 2018; Shen et al., 2021). In *Vitis davidii* (Roman. Du Caill.) Foëx. and red grape cultivars, UGT genes show high expression levels in colored fruit skins, whereas their expression is absent or greatly reduced in white-fruited varieties (Sun et al., 2016). In the present study, the spatiotemporal expression pattern of *RiUGT43* was closely associated with anthocyanin accumulation. qRT-PCR analysis showed that *RiUGT43* expression reached its highest level at the full-bloom stage (S3) and was barely detectable in roots, stems, and leaves, coinciding with the period of maximal anthocyanin accumulation. This spatial and developmental pattern further supports the positive transcriptional correlation between *RiUGT43* transcript abundance and anthocyanin accumulation.

Studies involving overexpression and gene silencing have been widely used to examine the correlation between transcript levels and phenotypic changes in various plant species. For example, overexpression of *Rd3GT1* and *Rd3GT6* from *Rhododendron delavayi* increased anthocyanin accumulation and enhanced flower coloration in transgenic tobacco, whereas silencing of *PeUFGT3* in *Phalaenopsis* reduced anthocyanin accumulation and caused petal fading (Chen et al., 2011; Sun et al., 2022). In addition, *PavUGT48* from sweet cherry was demonstrated to catalyze the glycosylation of cyanidin, generating cyanidin-3-*O*-glucoside and promoting fruit coloration (Feng et al., 2025). In sweet cherry, genome-wide identification of 187 UGT members, together with expression and co-expression network analyses, further highlighted the potential importance of *PavUGT48* in anthocyanin accumulation (Feng et al., 2025).

The *RiUGT43*-GFP fusion construct has been shown to express in *N. benthamiana* leaves. However, transient expression systems in petals remain technically challenging in many ornamental species. In transgenic *Limonium* flowers, green fluorescence could not be observed due to anthocyanin interference (Mercuri et al., 2001). Previous studies have shown that vacuolar anthocyanins can statically quench GFP fluorescence (Collings, 2019), and that inner filter effects as well as autofluorescence may interfere with GFP detection in plant materials (Su et al., 2005). In addition, protein extraction from pigment-rich petal tissues poses inherent difficulties for Western blot analysis. Polyphenols can form irreversible complexes with proteins, thereby interfering with detection (Loomis, 1974; Westermeier, 2006), and phenolic compounds severely interfere with protein quantification in pigment-rich tissues (Mattoo et al., 1987). In this study, evidence for exogenous RiUGT43 protein accumulation is lacking in *Rhododendron* petals. Therefore, the observed changes in anthocyanin content cannot be attributed to the catalytic activity of *RiUGT43*, and our conclusions are strictly limited to the positive transcriptional correlation between *RiUGT43* transcript abundance and anthocyanin accumulation. In future experiments, we will further investigate this using modified expression systems.

In the present study, transient overexpression and VIGS-mediated silencing assays were performed in red flowers of *R. indicum* ‘Huazhui’ to investigate the relationship between *RiUGT43* transcript level and anthocyanin accumulation. Although silencing of *RiUGT43* significantly reduced its transcript abundance (to 0.41-fold of the control), total anthocyanin content decreased only modestly (to 0.88-fold of the control, corresponding to a 12% reduction). This relatively mild phenotypic effect may reflect redundancy or compensation at the transcriptional or post-transcriptional level among other UGT family members-a common challenge in single-gene correlative analyses given the large number and high sequence similarity of UGT family members. Because total anthocyanin content was measured using the pH differential method, the present data do not establish the substrate specificity or glycosylation position of *RiUGT43*. Future studies will include targeted LC-MS analysis using authentic standards and *in vitro* enzymatic assays to test whether *RiUGT43* has glycosyltransferase activity and to determine its substrate specificity.

## 5 Conclusion

In conclusion, we identified 137 UGT family members in the *R. simsii* genome and classified them into 15 groups (A–O), providing a genomic framework for understanding UGT evolution in *Rhododendron*. The genome-level homolog of *RiUGT43* was assigned to Group E, and its preferential expression in flowers, particularly at the full-bloom stage of red flowers in *R. indicum* ‘Huazhui’, suggested its potential transcriptional association with pigmentation. Transient overexpression and VIGS assays in red petals of *R. indicum* ‘Huazhui’ demonstrated that overexpression of *RiUGT43* significantly increased its transcript abundance and total anthocyanin content, whereas silencing of *RiUGT43* reduced both its transcript level and anthocyanin accumulation, indicating that *RiUGT43* transcript abundance is positively correlated with anthocyanin accumulation. Localization and accumulation of the *RiUGT43*-GFP fusion protein were confirmed in *N. benthamiana* leaves. Collectively, our findings suggest that *RiUGT43* is transcriptionally associated with flower color regulation and provide candidate targets and molecular resources for future research and ornamental breeding aimed at improving flower color traits. However, due to the lack of direct protein-level evidence in petals, these conclusions are restricted to the transcriptional level.

## Data Availability

The original contributions presented in the study are included in the article/**Supplementary Material**. Further inquiries can be directed to the corresponding author.
