# Supplementary material for: Genome-wide analysis of the UGT gene family in Rhododendron and transcriptional correlation of RiUGT43 with anthocyanin accumulation
Source: Front Plant Sci. 2026 Sep 23;17:1957991. doi: 10.3389/fpls.2026.1957991 (PMC13642658; doi:10.3389/fpls.2026.1957991)
Supplement: Supplementary file 1 [file DataSheet1.zip › 附件/Supplementary Figures.docx]

**Supplementary Figures**

**Figure S1. Amino acid sequence alignment of the RiUGT43 transcript with RsUGT42–RsUGT44 homologous proteins.** Multiple sequence alignment between the RiUGT43 transcript (translated into amino acid sequence) identified from the *R. indicum* transcriptome and the RsUGT42, RsUGT43, and RsUGT44 proteins predicted from the *R. simsii* genome. Pairwise sequence Identity and Positives percentages are shown. *RiUGT43* exhibits 95.7% Identity with *RsUGT43*,83.7% Identity with *RsUGT43* and 82.7% Identity with *RsUGT44*. Indicating that *RiUGT43* is the orthologous transcript of *RsUGT43* in *R. indicum*.

**Figure S2. Conserved domain, transmembrane prediction, hydrophilicity, and signal peptide.**

**Figure S3. Secondary and tertiary structure prediction of *RiUGT43*.** (A) Predicted secondary structure showing α-helices (cylinders), β-strands (arrows), turns (lines) and coils (coils). (B, C) Predicted tertiary structure in two orientations. The protein adopts a canonical GT-B fold characteristic of the UGT family.

**Figure S4. Domain architecture and conserved motif analysis of the *RiUGT43* protein.** The UDP-glycosyltransferase domain (UDPGT domain) is located at amino acid residues 1–324, followed by the PSPG box (residues 417–460). Within the PSPG box, the highly conserved HCGWNS motif (residues 435–440) is highlighted in dark red. The full PSPG box sequence (417–460) is shown below, with the conserved motif underlined. The domain organization indicates that *RiUGT43* belongs to the UGT family and contains the characteristic PSPG motif essential for UDP-sugar binding and catalytic activity.

**Figure S5. Multiple sequence alignment of *RsUGT43* with *AtUGT79B2* and *AtUGT79B3*.** The boxed region indicates the conserved PSPG (plant secondary product glycosyltransferase) box domain.

**Figure S6. Western blot analysis of the recombinant RiUGT43 protein.** M, protein molecular weight marker (KDa).
